# The association between dietary intake of branched-chain amino acids and odds and severity of rheumatoid arthritis

**DOI:** 10.1038/s41598-024-56610-4

**Published:** 2024-03-15

**Authors:** Mohadeseh Soleimani Damaneh, Naheed Aryaeian, Shole Khajoenia, Leila Azadbakht, Fatemeh Sadat Hosseini-Baharanchi

**Affiliations:** 1 Department of Nutrition, School of Public Health, Iran University of Medical Sciences, Tehran, Iran; 2 Department of Clinical Science, Faculty of Medicine, Medical Science University of Jiroft, Jiroft, Iran; 3 Department of Community Nutrition, School of Nutritional Sciences and Dietetics, Tehran University of Medical Sciences, Tehran, Iran; 4 Diabetes Research Center, Endocrinology and Metabolism Clinical Sciences Institute, Tehran University of Medical Sciences, Tehran, Iran; 5 Department of Biostatistics, School of Public Health, Iran University of Medical Sciences, Tehran, Iran

**Keywords:** Rheumatic diseases, Risk factors

## Abstract

This case–control study investigated the link between dietary branched-chain amino acids (BCAAs) and the risk and severity of rheumatoid arthritis (RA). We assessed dietary BCAA intake in 95 RA patients and 190 matched controls using a food frequency questionnaire. We also assessed the disease severity using the disease activity score 28 (DAS-28), ESR, VAS, morning stiffness, and tender and swollen joints. Higher BCAA intake, expressed as a percentage of total protein, was significantly associated with increased risk of RA for total BCAAs (OR 2.14, 95% CI 1.53–3.00, *P* < 0.001), leucine (OR 2.40, 95% CI 1.70–3.38, *P* < 0.001), isoleucine (OR 2.04, 95% CI 1.46–2.85, *P* < 0.001), and valine (OR 1.87, 95% CI 1.35–2.59, *P* < 0.001). These associations remained significant even after adjusting for potential confounders (*P* < 0.001). However, BCAA intake did not show any significant association with RA severity in either crude or multivariate models (*P* > 0.05). Our findings suggest that higher dietary BCAA intake may contribute to the development of RA, but further research is needed to confirm these observations and explore the underlying mechanisms.

## Introduction

Rheumatoid arthritis (RA), a chronic autoimmune disease, inflames the synovial membrane in the joints, leading to pain, swelling, stiffness, and progressive joint damage that can ultimately limit mobility^1^. Approximately 0.5–1% of the worldwide population and 0.37% of Iranians are affected by RA, and women are affected 2 to 3 times more often than men^2,3^. Although the exact etiology of RA is not yet well-known, studies have shown that some environmental factors, such as obesity, smoking, and changes in the gut microbiome, can increase the risk of developing RA^4^. Diet is also one of the most important environmental factors that may increase or decrease the risk of developing RA and also affect disease activity by modulating the immune system^5^. It has shown that dietary intakes of fish, mushroom, beans, olive oil, citrus fruits, and vegetables may have independent protective effects on RA development or severity^6,7^. On the contrary, it has been discovered that Western dietary patterns, which include high consumption of sweet snacks, high-fat meat, refined grains, and high-fat dairy products, can be directly associated with a higher risk of RA^8^. In addition, a prospective study revealed that a higher intake of red meat and total protein was associated with a higher risk of RA^9^. Also, the positive effect of plant-based proteins in reducing disease activity and improving metabolic status in people with RA has been reported^10–12^. The connection between branched-chain amino acids (BCAAs) and chronic diseases has recently received increasing attention. BCAAs (leucine, valine, and isoleucine) are essential amino acids that the body cannot produce and must be obtained from food^13^. It has been reported that elevated plasma BCAA levels may be related to increased inflammation and oxidative stress levels^14^, which are associated with the pathogenesis of different health conditions including RA^15–17^. Several plasma-based prior studies have also demonstrated a positive link between plasma BCAA concentrations and the risk of metabolic conditions such as cardiovascular diseases (CVD), type 2 diabetes (T2D), and insulin resistance^18,19^. However, the interactions between BCAAs and health are complex, and some other studies present conflicting results^20,21^. There is still no study evaluating the relationship between dietary BCAAs and RA. Therefore, this case–control study examined the relationship between dietary BCAA intake and RA risk. We also evaluated the relationship between BCAA intake and some clinical markers of disease severity in RA patients.

## Methods

### Study participants

This was a 2:1 age (± 5 years) and gender matched case–control study carried out from February to May 2022 in Kerman, Iran. It included 95 patients with RA and 190 healthy controls aged 18–80 years. Participants were recruited using a convenient sampling method. RA patients were selected consecutively from those who were referred to the rheumatology clinic at Besaat Clinic in Kerman, Iran. For each case, we enrolled 2:1 matched healthy control according to their age (± 5 years) and gender from the companions of patients attending other departments within the same clinic. RA diagnosis was made by a rheumatologist based on the American College of Rheumatology 2010 classification criteria. RA onset was defined as the day of diagnosis, as stated in participant medical records. Only RA cases with a maximum of one year since diagnosis were included. The current study was carried out according to the Declaration of Helsinki, and the study protocol was approved by the Ethics Committee of the Iran University of Medical Sciences (IR.IUMS.REC.1400.859). Each participant gave written consent after receiving information about the study's aims and procedures.

### Inclusion criteria

Adult patients (18–80 years) who met the American College of Rheumatology (ACR) criteria for a clinical diagnosis of RA by a rheumatologist were included in the study^22^. The inclusion criteria for subjects in the control group were adults between 18 and 80 without joint or connective tissue disorders.

### Exclusion criteria

Subjects in both case and control groups with a history of alcohol consumption, disease (e.g., hepatic or renal disease, CVD, T2D, thyroid abnormalities, hyperlipidemia, and cancer), food allergies, adherence to special diets (vegetarian, weight gain, very-low-energy, or ketogenic diets), food prohibition, consumption of dietary supplements, taking certain medicines (except anti-inflammatory drugs for patients) during the year before the interview; and reported implausible caloric intakes (< 800 or > 4200 kcal/day) were excluded. We also excluded participants who did not answer more than 35 items of the FFQ.

### Dietary assessment

To assess participants' dietary intake, we utilized a valid and reliable 168-item semi-quantitative food frequency questionnaire (FFQ)^23^. Individuals were asked to specify their frequency of consumption for each dietary component during face-to-face interviews conducted by an experienced dietician, listing them as daily, weekly, monthly, or yearly. The standard serving sizes of consumed foods reported in household measures were then converted into grams. Then, the Nutritionist IV program [Nutritional Database Manager 4.0.1] was used to analyze food data for energy and nutrients. Based on the USDA food composition table, information on the energy, macro-, micronutrient, and amino acid content, including leucine, isoleucine, and valine of each food, was derived. Iranian FCT17 was used for local food not covered by USDA FCT^24^. Daily intake of total BCAAs was calculated for each participant by summing their intake of leucine, isoleucine, and valine, based on their usual consumption of foods containing these amino acids. Evidence on the validity of FFQs for estimating BCAA intake is limited. However, a previous validation study found that FFQs were able to accurately assess total protein intake. The individual branch chain amino acids valine, leucine, and isoleucine were also highly correlated with each other^25^.

### Determination of RA disease activity

The DAS28-ESR (Disease activity scores in 28 joints calculated with Erythrocyte Sedimentation Rates) was used to assess disease activity. DAS28 is derived from four components, including swollen joint count (SJC), tender joint count (TJC) of 28 joints, ESR(mm/h) as a marker of systemic inflammation, and global health assessment, which is indicated by the visual analog scale (VAS)^26^. DAS-28 scores are categorized as follows: remission (≤ 2.6), low (> 2.6 and ≤ 3.2), moderate (> 3.2 and ≤ 5.1), and high (> 5.1) disease activity. We used the clinical information in the patient’s medical records to determine the ESR measured the previous month. The period from when patients were awake until their pain improved was used to assess the length of Morning Stiffness (MS). We used a Visual Analogue Scale (VAS) (0–10 mm pain scale: 0 = none and 10 = intolerable) to measure pain intensity. This scale has been proven valid in previous studies^27^.

### Assessment of other variables

The required data on other variables, including demographics (age, gender, education), smoking status, drug use (Biologic and Synthetic disease-modifying anti-rheumatic drugs (DMARDs), corticosteroids, and non-steroidal anti-inflammatory drugs (NSAIDs)), and past medical history, were collected through a general information questionnaire. Physical activity levels were determined using the Persian version of the International Physical Activity Questionnaire (IPAQ)^28^. In light clothing and without shoes, body weight was measured using a digital scale. The standing height was measured without shoes to the nearest 0.5 cm. The Body Mass Index (BMI) was calculated as body weight (kg)/height (m)^2^.

### Statistical analysis

Statistical analyses were conducted by SPSS software, version 26 (SPSS Inc). The results were reported as frequency (%) and mean ± SD for qualitative and quantitative data. We used Kolmogorov–Smirnov analysis to assess the normality. The chi-square and independent samples T-test for categorical and continuous variables were employed to compare the two groups, respectively. We used one-way ANOVA for continuous and chi-square tests for categorical variables to compare general characteristics and dietary intakes across tertiles of BCAAs. Because previous studies have shown a very high correlation between BCAA and total protein intake, BCAA intake was expressed as a percentage of total protein intake. The distribution of clinical parameters of RA disease activity across tertiles of the percentage of BCAAs intake of total protein intake was also determined by chi-square tests and ANOVA. Multivariable conditional logistic regression was used to estimate the odds ratios (ORs) and 95% confidence intervals (CIs) for the associations between dietary intake of BCAAs (independent variables) with the risk of RA (dependent variable). ORs and 95% CIs were obtained according to the percentile of BCAAs intake in the crude and multivariable-adjusted models as follows: Model 1 adjusted for BMI, education, smoking, hypertension, and physical activity, and Model 2 adjusted for covariates in Model 1 as well as for energy. Simple and multiple linear regressions were performed to assess the associations between BCAA intake as a continuous variable and DAS-28, VAS, early morning stiffness (min), ESR, and the number of tender and swollen joints as outcomes and BCAA intake as a continuous variable. Multiple linear regressions were adjusted in Step 2 for covariates, including age, sex, BMI, education, smoking, drug use, hypertension, and physical activity. In Step 3, Step 2, plus energy intake. A *P*-value < 0.05 was regarded as significant for all statistical analyses.

## Results

Table 1 presents the general characteristics of the study participants. The mean age of the RA patients was 48.86 ± 9.41 years, and the mean age of the healthy controls was 47.46 ± 9.30 years. Female participants constituted 77.89% of the participants in both groups. The mean weight of participants with RA was significantly lower than that of participants in the control group (*P* < 0.001). However, the mean BMI of the two groups was not significantly different (*P* = 0.06). Protein intake was significantly higher in the control group than in the RA group (*P* < 0.05). Fat intake, on the other hand, was significantly higher in the RA group than in the control group (*P* < 0.05). The total intake of BCAA for the case group was 16.86 ± 3.3% protein and for the control group it was 14.47 ± 2.94%. The percentage of total BCAAs, isoleucine, leucine, and valine intake of total protein intake were significantly higher in the RA group than in the control group (*P* < 0.001).

**Table 1:** Characteristics of study participants across case and control groups.

	Case (n = 95)	Control (n = 190)	*P*-value†
Age (year)	48.86 ± 9.41	47.46 ± 9. 3	0.12
Gender [n, (%)]			
Female, n (%)	74 (77.89)	148 (77.89)	1
Weight (kg)	65.25 ± 13.2	73.81 ± 12.72	< 0.001
BMI (kg/m2 )	25.23 ± 4.92	26.36 ± 4.34	0.06
Smoker, n (%)	5 (5.26)	17 (8.95)	0.35
Educational status [n, (%)]			
< 12 years, n (%)	76 (80)	146 (76.84)	0.54
> 12 years, n (%)	19 (20)	44 (23.16)	
Physical activity level [n, (%)]			
Light, n (%)	73 (76.84)	146 (76.84)	0.48
Moderate, n (%)	22 (23.16)	36 (18.95)	
Heavy, n (%)	0	8 (4.21)	
Hypertension, n (%)	14 (14.74)	34 (17.89)	0.55
Dietary BCAAs			
Total BCAAs (g/day)	11.23 ± 3.43	10.52 ± 2.62	0.06
Total BCAAs (% total protein intake/day)	16.86 ± 3.3	14.47 ± 2.94	< 0.001
Isoleucine (g/day)	2.98 ± 9.79	2.73 ± 6.91	0.21
Isoleucine (% total protein intake/day)	4.45 ± 0.93	3.76 ± 0.75	< 0.001
Leucine (g/day)	4.78 ± 1.49	4.60 ± 1.46	0.63
Leucine (% total protein intake/day)	7.3 ± 2.94	6.33 ± 1.28	< 0.001
Valine (g/day)	3.38 ± 1.03	3.2 ± 0.8	0.97
Valine (% total protein intake/day)	5.07 ± 1.01	4.40 ± 0.91	< 0.001
Energy intake (kcal/day)	2127.35 ± 552.43	2189.28 ± 532.54	0.36
Protein(g/day)	67.9 ± 20.44	74.33 ± 18.93	0.01
Fat(g/day)	73.93 ± 30.57	61.99 ± 25.73	< 0.001
Carbohydrate(g/day)	305.74 ± 85.34	337.03 ± 92.39	0.18
Fiber(g/day)	18.56 ± 6.37	17.7 ± 6.13	0.27
Drug use [n, (%)]			
Synthetic-DMARDs	87 (91.6)	–	–
Biologic-DMARDs	36 (37.9)	–	–
Corticosteroids	80 (84.21)	–	–
NSAIDs	28 (29.5)	–	–

Data are presented as means ± standard deviation or number (percent). BCAAs, Branched-chain amino acids; BMI, Body mass index; DMARDs, Disease-modifying anti-rheumatic drugs; NSAIDs, Non-steroidal anti-inflammatory drugs. †*P* values were obtained by the chi-square test and independent samples T-test for categorical and continuous variables, respectively.

Table 2 illustrates the general characteristics of the study population across tertiles of percentage of BCAA of total protein intakes. Participants in the highest tertile of total BCAA intake had significantly higher intakes of energy, protein, fat, carbohydrate, and fiber than participants in the lowest tertile (all *P* for trend < 0.05). Additionally, participants in the highest tertile of total BCAA intake were older than those in the lowest tertile (*P* for trend < 0.05). No other variables were significantly different across tertiles of BCAAs.

**Table 2:** General characteristics of study participants across tertiles of BCAAs.

Variables	Tertiles of BCAAs (% total protein intake)			
	T1 (< 14.45%)	T2 (14.45–16.87%)	T3 (> 16.87%)	*P*†
n	95/285	95/285	95/285	
Age (year)	45.09 ± 9.36	47.61 ± 8.83	51.08 ± 8.92	< 0.0001
Female, n (%)	70 (73.7)	74 (77.9)	78 (82.1)	0.37
Weight (kg)	73.20 ± 12.66	70.94 ± 13.54	68.72 ± 13.96	0.07
BMI (kg/m2 )	26.31 ± 3.92	25.96 ± 4.26	25.66 ± 5.41	0.62
Smoker, n (%)	8 (8.4)	7 (7.4)	7 (7.4)	0.95
Educational status				0.94
< 12 years, n (%)	73 (76.8)	74 (77.9)	75 (78.9)	
> 12 years, n (%)	22 (23.2)	21 (22.1)	20 (21.1)	
Physical activity level				0.06
Light	68 (71.6)	79 (83.2)	72 (75.8)	
Moderate	22 (23.2)	13 (13.7)	23 (24.2)	
Heavy	5 (5.3)	3 (3.2)	0 (0)	
Hypertension, n (%)	17 (17.9)	15 (15.8)	16 (16.8)	0.92
Energy intake (kcal/day)	2287.72 ± 619.17	2197.96 ± 516.13	2020.21 ± 436.76	0.002
Protein(g/day)	77.33 ± 23.14	72.79 ± 18.81	66.42 ± 14.67	< 0.0001
Fat(g/day)	68.73 ± 32.53	68.98 ± 28.49	60.18 ± 20.99	0.04
Carbohydrate(g/day)	351.33 ± 105.14	329.41 ± 91.89	299.05 ± 64.9	< 0.0001
Fiber(g/day)	17.67 ± 7.22	19.35 ± 6.62	16.92 ± 4.18	0.02

BCAA intake was expressed as percent protein intake; †Obtained from ANOVA for continuous variables and the chi-squared test for categorical variables. BCAAs, Branched-chain amino acids; BMI, Body mass index; DMARDs, Disease-modifying anti-rheumatic drugs; NSAIDs, Non-steroidal anti-inflammatory drugs.

Table 3 shows the odds ratio (95% CI) and β for risk of RA based on total BCAAs, valine, leucine, and isoleucine intake. According to the continuous BCAAs intake (% total protein intake), a significant positive association was found between total BCAA (OR 2.14; 95% CI 1.53–3.0, *P* < 0.001), leucine (OR 2.4; 95% CI 1.7–3.38, *P* < 0.001), isoleucine (OR 2.04; 95% CI 1.46–2.85, *P* < 0.001), and valine (OR 1.87; 95% CI 1.35–2.59, *P* < 0.001), and the risk of developing RA; These associations remained significant even after controlling for the potential confounders (*P* < 0.001).

**Table 3:** Odds ratios (95% confidence interval) of RA risk according to the tertiles of total BCAAs, Isoleucine, Leucine, and Valine intake.

Variable	OR (CI)	β	*P*_value_
Total BCAAs (% total protein intake)			
Crude	2.14 (1.53–3.0)	0.76	< 0.001
Model 1^a^	2.12 (1.50–2.98)	0.75	< 0.001
Model 2^b^	2.15 (1.51–3.05)	0.76	< 0.001
Isoleucine (% total protein intake)			
Crude	2.40 (1.7–3.38)	0.87	< 0.001
Model 1^a^	2.37 (1.67–3.36)	0.86	< 0.001
Model 2^b^	2.48 (1.71–3.59)	0.91	< 0.001
Leucine (% total protein intake)			
Crude	2.04 (1.46–2.85)	0.71	< 0.001
Model 1^a^	2.01 (1.43–2.83)	0.70	< 0.001
Model 2^b^	2.04 (1.43–2.89)	0.71	< 0.001
Valine (% total protein intake)			
Crude	1.87 (1.35–2.59)	0.63	< 0.001
Model 1^a^	1.84 (1.31–2.57)	0.61	< 0.001
Model 2^b^	1.85 (1.31–2.62)	0.62	< 0.001

Total and individual BCAA intake was expressed as percent protein intake; Data were analyzed by multivariate conditional logistic regression model (cox regression) using enter method. Data are OR (95% CI). ^a^Model 1 was adjusted for BMI, education, smoking, hypertension, and physical activity. ^b^Model 2 was adjusted for Model 1 plus energy intake.

Table 4 presents the clinical markers of disease activity in RA patients across tertiles of BCAAs (% total protein intake). There were no significant differences between ESR (*P* trend = 0.36), DAS28 (*P* trend = 0.84), VAS (*P* trend = 0.92), duration of early morning stiffness (*P* trend = 0.13), and the number of tenders and swollen joints (*P* trend = 0.87 and *P* trend = 0.28, respectively) across tertiles of total BCAA intake.

**Table 4:** Distribution of clinical markers of RA across tertiles of BCAAs.

Variable	Total	Tertiles of BCAAs (% total protein intake)			*P*_Trend†_
		T1	T2	T3	
n	95	10/95	44/95	41/95	
EMS (min)	13.67 ± 19.1	9.6 ± 20.01	10.40 ± 13.69	18.17 ± 23.01	0.13
DAS28	2.7 ± 1.10	2.85 ± 1.0	2.76 ± 1.18	2.65 ± 1.13	0.84
TJC	0.95 ± 1.31	1.20 ± 1.61	0.95 ± 1.32	0.97 ± 1.25	0.87
SJC	2.47 ± 2.36	2.40 ± 1.95	2.09 ± 1.97	2.90 ± 2.78	0.28
ESR (mm/h)	20.15 ± 19.95	20.60 ± 24.14	23.09 ± 23.73	16.90 ± 13.34	0.36
VAS(mm)	40.21 ± 20.32	40.20 ± 20.65	40.11 ± 20.33	40.31 ± 20.29	0.92

BCAA intake was expressed as percent protein intake; †The significant difference based on One-way ANOVA (*P* < .05). EMS, Early morning stiffness; DAS28, Disease Activity Score-28; TJC, Tender joint count; SJC, Swollen joint count; ESR, Erythrocyte sedimentation rate; VAS, visual analog scale.

The results of simple and multiple linear regressions to assess the association of dietary BCAA consumption with clinical markers of RA are presented in Table 5. In simple linear regression, no significant associations were found between the clinical markers and total and individual BCAA intake. This remained true after adjusting for age, sex, BMI, education, smoking, drug use, hypertension, physical activity, and energy intake in separate steps (*P* > 0.05).

**Table 5:** Association between total and individual BCAAs intake and clinical markers of RA.

	Total BCAAs (% total protein intake)		Isoleucine (% total protein intake)		Leucine (% total protein intake)		Valine (% total protein intake)	
	β (95% CI)	*P*-value	β (95% CI)	*P*-value	β (95% CI)	*P*-value	β (95% CI)	*P*-value
EMS (min)								
Step 1ͣ	0.47 (− 0.71 to 1.65)	0.43	1.12 (− 3.10 to 5.34)	0.05	1.26 (− 1.41 to 3.93)	0.35	1.76 (− 2.08 to 5.60)	0.36
Step 2^b^†	0.008 (− 1.22 to 1.24)	0.99	0.03 (− 4.28 to 4.34)	0.98	0.11 (− 2.67 to 2.90)	0.93	0.07 (− 3.97 to 4.12)	0.97
Step 3^b^†	− 0.45 (− 1.75 to 0.83)	0.48	− 1.12 (− 5.54 to 3.28)	0.61	− 1.0 (− 3.96 to 1.95)	0.50	− 1.62 (− 5.93 to 2.69)	0.45
DAS28								
Step 1ͣ	− 0.03 (− 0.09 to 0.03)	0.37	− 0.14 (− 0.38 to 0.10)	0.25	− 0.04 (− 0.19 to 0.11)	0.58	− 0.09 (− 0.31 to 0.13)	0.42
Step 2^b^	− 0.05 (− 0.11 to 0.002)	0.05	− 0.19 (− 0.40 to 0.02)	0.07	− 0.11 (− 0.25 to 0.02)	0.10	− 0.19 (− 0.39 to 0.001)	0.05
Step 3^b^	− 0.05 (− 0.12 to 0.006)	0.07	− 0.18 (− 0.40 to 0.03)	0.09	− 0.11 (− 0.26 to 0.03)	0.14	− 0.20 (− 0.42 to 0.01)	0.06
TJC								
Step 1ͣ	0.003 (− 0.07 to 0.08)	0.94	0.01 (− 0.27 to 0.30)	0.92	0.02 (− 0.16 to 0.20)	0.81	− 0.006 (− 0.27 to 0.26)	0.96
Step 2^b^	− 0.02 (− 0.10 to 0.05)	0.58	− 0.05 (− 0.33 to 0.23)	0.72	− 0.03 (− 0.22 to 0.14)	0.68	− 0.09 (− 0.36 to 0.16)	0.47
Step 3^b^	− 0.01 (− 0.10 to 0.07)	0.72	− 0.03 (− 0.32 to 0.26)	0.83	− 0.01 (− 0.21 to 0.17)	0.84	− 0.07 (− 0.36 to 0.21)	0.59
SJC								
Step 1ͣ	0.01 (− 0.12 to 0.16)	0.79	0.05 (− 0.47 to 0.57)	0.84	0.07 (− 0.25 to 0.40)	0.66	0.06 (− 0.41 to 0.54)	0.79
Step 2^b^	− 0.05 (− 0.19 to 0.09)	0.48	− 0.11 (− 0.61 to 0.37)	0.63	− 0.09 (− 0.41 to 0.22)	0.54	− 0.18 (− 0.65 to 0.28)	0.43
Step 3^b^	− 0.03 (− 0.18 to 0.11)	0.64	− 0.07 (− 0.59 to 0.44)	0.77	− 0.06 (− 0.41 to 0.28)	0.72	− 0.13 (− 0.64 to 0.37)	0.59
ESR (mm/h)								
Step 1ͣ	− 0.54 (− 1.78 to 0.68)	0.38	− 2.53 (− 6.92 to 1.85)	0.25	− 0.87 (− 3.67 to 1.92)	0.53	− 1.60 (− 5.62 to 2.41)	0.43
Step 2^b^	− 0.85 (− 2.13 to 0.41)	0.18	− 3.32 (− 7.78 to 1.12)	0.14	− 1.58 (− 4.48 to 1.30)	0.27	− 2.76 (− 6.95 to 1.42)	0.19
Step 3^b^	− 0.51 (− 1.87 to 0.84)	0.45	− 2.41 (− 7.01 to 2.18)	0.29	− 0.70 (− 3.81 to 2.39)	0.65	− 1.52 (− 6.05 to 2.99)	0.50
VAS								
Step 1ͣ	− 0.03 (− 0.17 to 0.11)	0.65	− 0.04 (− 0.56 to 0.46)	0.85	− 0.04 (− 0.37 to 0.28)	0.78	− 0.15 (− 0.61 to 0.32)	0.52
Step 2^b^	− 0.09 (− 0.23 to 0.04)	0.16	− 0.22 (− 0.71 to 0.26)	0.36	− 0.19 (− 0.51 to 0.11)	0.21	− 0.38 (− 0.83 to 0.06)	0.09
Step 3^b^	− 0.04 (− 0.18 to 0.10)	0.58	− 0.06 (− 0.55 to 0.42)	0.79	− 0.05 (− 0.38 to 0.27)	0.74	− 0.18 (− 0.67 to 0.29)	0.43

^a^Simple linear regression analysis. ^b^Multiple linear regression analysis. †Step 1 was crude model, Step 2 was adjusted for age, sex, BMI, education, smoking, hypertention, drug use, and physical activity; Step 3 was adjusted for Step 2 plus energy intaake.

## Discussion

In this case–control study, we found a positive association between dietary intake of BCAAs and the risk of RA. In individual analysis of the three BCAAs, we also observed that a higher intake of valine, leucine, and isoleucine were associated with a higher risk of RA. However, there was no significant association between total and individual dietary BCAA intake and disease activity parameters, including DAS28, ESR, pain VAS, morning stiffness, and the number of tender and swollen joints.

RA is a systemic autoimmune inflammatory disorder that primarily affects the joints and leads to bone erosion and disability^1^. Although the exact pathogenesis of RA is unknown, genetic and environmental factors affect disease progression^4^. Several studies have shown that diet can be an environmental factor associated with RA^5^. A large prospective study showed that higher consumption of red meat, meat by-products, and total proteins was associated with a higher risk of inflammatory polyarthritis^29^. The relationship between chronic diseases and BCAAs has recently received increasing attention. BCAAs are essential amino acids that must be included in the diet and are crucial for protein synthesis. They also function as signaling molecules in metabolic pathways that regulate glucose and energy homeostasis and immunity^30^. Previous studies showed a strong correlation between plasma BCAA levels and amino acid intake as measured by the FFQ and food records^31^. Several human studies have reported a positive association between elevated plasma BCAAs and insulin resistance, cardiometabolic disorders, T2D, and obesity^18,32,33^. Some studies have reported that circulating BCAA levels decrease with body weight loss after dietary intervention or weight loss surgery^34^. Moreover, the Nurses’ Health Study II cohort conducted from 1996 to 1999 showed that higher dietary intakes and circulating levels of total and individual BCAAs are associated with an increased risk of progression from GDM to T2D later, and BCAAs have been suggested as a critical predictor of future diabetes^31^. In a case–control serum-based study, Zhai et al. found that the ratio of BCAAs to histidine was significantly associated with knee osteoarthritis. This ratio was described as a novel biomarker in these patients^35^. However, there is a complex interaction between BCAAs and health, and some studies have yielded conflicting findings. A dose–response meta-analysis in 2021 showed that red meat, poultry, and dairy intake as the primary dietary sources of BCAAs were not associated with the risk of RA^36^. In addition, a cross-sectional study of young Northern Chinese adults found that a higher proportion of BCAAs was inversely associated with the prevalence of overweight/obesity, abdominal obesity, postprandial glucose tolerance, and inflammatory status^21^. These contradictory results might be due to differences in dietary habits and patterns, which are particular for people from various regions; for instance, the primary dietary pattern in Northern Chinese is carbohydrate-rich^37^. Moreover, a cross-sectional study of 298 healthy individuals was conducted to determine which dietary pattern is linked to plasma BCAA concentrations. The researchers discovered that the dietary pattern that was linked to plasma BCAA concentrations is similar to a Western diet high in animal-based protein. The protein in the Western diet has been reported to consist of > 20% BCAAs^38^. Indeed, evidence shows that Western dietary patterns are more in line with BCAA-explained dietary patterns. The complexity of diet is probably another factor contributing to the contradictory findings. The diet combines numerous nutrients, which differ significantly between people and cultures^39^.

In line to our findings about the significant association between the risk of RA and individual BCAAs, Mirmiran et al. in a population-based prospective cohort study found that higher dietary intakes of total BCAAs, particularly valine, were positively associated with a higher risk of hypertension^40^. Additionally, an animal study by Deyang Yu et al. found that reducing dietary isoleucine and/or valine corrected some metabolic abnormalities, including insulin resistance, hyperglycemia, and hepatic steatosis in mice^41^. Also, a prospective cohort study by Asghari et al. demonstrated that a higher intake of BCAAs, especially leucine and valine, increases the risk of insulin resistance in adults^42^.

This study also investigated the association between BCAA intake and some clinical parameters related to disease activity, including DAS28, ESR, pain VAS, morning stiffness, and the number of tender and swollen joints. Dietary components can affect disease activity in RA by directly or indirectly modulating the immune system. There are few reports on diet and nutrients' effects on RA activity. In a study on RA patients, Tandorost et al. found a positive relationship between the Dietary Inflammatory Index (DII) score (a pro-inflammatory diet) and DAS28^43^. Additionally, in RA individuals with low to moderate disease activity, a 16-week lifestyle program centered on a whole plant-based diet, physical activity, and stress management improved metabolic status and decreased disease activity^10^. Following a Mediterranean diet, with limited meat consumption, has also shown beneficial effects on clinical disease progression in RA patients by reducing DAS28, pain, and tender joints^44,45^. A Mediterranean diet intervention of 12 weeks could improve general health and decrease tender joints, but it was ineffective in improving physical function or morning stiffness^15^. The lack of association between BCAA intake and disease activity in our study may be due to the low disease severity in the majority of participants, as measured by the investigated parameters.

A few potential mechanisms could help to explain our findings regarding the association between dietary BCAA intake and an increased risk of RA and disease activity. Since BCAA are essential amino acids, a high level of free BCAAs may implicate high dietary intakes or increase protein degradation rates. After collagen was destroyed in arthritis-related inflamed joints, high amounts of BCAAs were found in the synovial fluid^46,47^. Alternatively, BCAAs by increasing the production of inflammatory cytokines, may lead to increased destruction of joint collagen^14^. In addition, High BCAA blood levels by increasing the production of more reactive oxygen species (ROS) and the activation of redox-sensitive transcription factor NF-κB can promote the activation of circulating peripheral blood mononuclear cells (PBMCs), which is paralleled to over-expression of the pro-inflammatory cytokines, such as tumor necrosis factor-α (TNF-α), interferon-ɣ, Interleukin-1β, and interleukin-6^14^. The etiology and progression of RA are significantly influenced by inflammatory cytokines^16^. In a large cross-sectional study (on 19,472 women), higher plasma BCAAs concentrations were correlated with adverse profiles of inflammatory biomarkers^48^, which could also be associated with increased disease activity in RA. Therefore, elevated BCAA levels could be related to the inflammatory process observed in pathological conditions, including RA^49^. Another possible mechanism may be related to the effect of diet on oxidative stress levels. Oxidative stress is a significant factor in the development of RA^50^. Excessive free radical generation, not an impaired antioxidant activity, is the leading cause of the oxidative stress associated with RA^16^. The imbalanced production of ROS is one of the most important mechanisms involved in different pathological conditions related to oxidative stress. The primary sources of ROS include NADPH oxidase and mitochondria. BCAAs induce ROS formation in PBMCs by activating two catalytic subunits of NADPH oxidase (NOX-1 and NOX-2)^51^. In addition, BCAAs increase the production of ROS in mitochondria, resulting in oxidative damage and mitochondrial dysfunction . The oxidative stress process is strongly related to inflammation, accelerated joint deterioration, and increased disease activity in RA patients^52^. Therefore, through increased oxidative stress, a diet high in BCAAs is probably associated with a higher risk of RA and disease activity. However, there are some inconsistent studies^53–55^. A study conducted on diabetic rats found that BCAAs reduce the levels of ROS. In another study, the administration of BCAAs could prevent the progression of non-alcoholic steatohepatitis (NASH) by reducing ROS^56^. However, human study differs from animal research regarding BCAA tolerance dose, BCAAs metabolism, and oxidative stress status. The major strengths of this study include adjusting for a wide range of possible confounders in statistical analysis and being the first investigation to examine the relation between BCAA intake and RA and some clinical parameters related to the disease activity. Our study also had some limitations. We could not determine the relationship between dietary intake and circulating BCAA levels because we did not have information on blood amino acid levels, which might limit the interpretability of our findings. We cannot differentiate between cause and effect in this study due to the case–control design. Because the recruitment of respondents was by convenience sampling, the generalizability to the entire Iranian RA population may be limited. Although a validated FFQ was used for dietary intakes assessment, the questionnaire was not validated for BCAAs intake. Therefore, to confirm our findings, more research is needed.

In conclusion, this case–control study of Iranian adults found a positive correlation between higher dietary intakes of BCAAs and the risk of RA. These findings suggest that a diet high in BCAAs may play a role in the development of RA. However, further studies are needed to investigate the potential roles of dietary BCAAs in RA and to confirm these findings.

## Data Availability

The datasets used and analyzed during the current study are available from the corresponding author upon reasonable request.

## Abbreviations

BCAAs: Branched-chain amino acids
OR: Odds ratio
CI: Confidence interval
RA: Rheumatoid arthritis
DAS28: Disease activity scores-28
VAS: Visual analog scale
ESR: Erythrocyte sedimentation rate
SD: Standard deviation
FFQ: Food frequency questionnaire
IPAQ: International Physical Activity Questionnaire
BMI: Body mass index
ACR: American College of Rheumatology
SJC: Swollen joint count
TJC: Tender joint count
ROS: Reactive oxygen species
PBMCs: Peripheral blood mononuclear cells
TNF-α: Tumor necrosis factor-α
EMS: Early morning stiffness
DMARDs: Disease-modifying anti-rheumatic drugs
NSAIDs: Non-steroidal anti-inflammatory drugs
